# Identification of microRNAs and relative target genes in *Moringa oleifera* leaf and callus

**DOI:** 10.1038/s41598-019-51100-4

**Published:** 2019-10-22

**Authors:** Stefano Pirrò, Ivana Matic, Arianna Guidi, Letizia Zanella, Angelo Gismondi, Rosella Cicconi, Roberta Bernardini, Vittorio Colizzi, Antonella Canini, Maurizio Mattei, Andrea Galgani

**Affiliations:** 1 Mir-Nat s.r.l., Rome, 00133 Italy; 2 Bioinformatics Unit, Centre for Molecular Oncology, Barts Cancer Institute, Queen Mary University London, London, EC1M 6BQ UK; 3 Department of Biology, University of Rome Tor Vergata, Rome, Italy; 4 CIMETA, University of Rome Tor Vergata, Rome, Italy

**Keywords:** Epigenetics analysis, Gene regulatory networks, High-throughput screening, Plant genetics, Plant molecular biology

## Abstract

MicroRNAs, a class of small, non-coding RNAs, play important roles in plant growth, development and stress response by negatively regulating gene expression. *Moringa oleifera* Lam. plant has many medical and nutritional uses; however, little attention has been dedicated to its potential for the bio production of active compounds. In this study, 431 conserved and 392 novel microRNA families were identified and 9 novel small RNA libraries constructed from leaf, and cold stress treated callus, using high-throughput sequencing technology. Based on the *M. oleifera* genome, the microRNA repertoire of the seed was re-evaluated. qRT-PCR analysis confirmed the expression pattern of 11 conserved microRNAs in all groups. MicroRNA159 was found to be the most abundant conserved microRNA in leaf and callus, while microRNA393 was most abundantly expressed in the seed. The majority of predicted microRNA target genes were transcriptional factors involved in plant reproduction, growth/development and abiotic/biotic stress response. In conclusion, this is the first comprehensive analysis of microRNAs in *M. oleifera* leaf and callus which represents an important addition to the existing *M. oleifera* seed microRNA database and allows for possible exploitation of plant microRNAs induced with abiotic stress, as a tool for bio-enrichment with pharmacologically important phytochemicals.

## Introduction

*Moringa oleifera* Lam. is an arboreal medicinal plant of Moringaceae family, popularly called the “miracle tree”, widely distributed in the subtropical belt^1^. Leaves, fruits, flowers and seeds of this plant are used as highly nutritious foods in many countries, particularly in India and Africa^2^. It has been reported that the leaves of *M. oleifera* constitute a source of β-carotene, proteins, vitamin C, calcium, potassium, and may act as a source of antioxidants. Several experiments have demonstrated the ability of *M. oleifera* leaves to protect the body and the cells from oxidative stress linked to cancer and degenerative diseases^3^. Furthermore, *M. oleifera* is rich in anti-inflammatory molecules, effective in immune protection, thanks to the presence of quercetin and caffeoylquinic acid^1,4^. Authoritative studies have shown that *M. oleifera* contains 46 antioxidants and 36 anti-inflammatory molecules, in addition to the omega-3-6-9 fatty acids^4,5^.

Recently, plant tissue and cell suspension cultures have been investigated by biotechnological methods in order to provide a promising bioproduction platform for desired natural products. Medicinal plant *in vitro* culture consists of free cells or small groups of cells obtained from medicinal plant callus cultured in a liquid medium. This suspension can produce a massive standardized yield of secondary metabolites for pharmaceutical use, independently from geographical, environmental and seasonal factors^6–9^.

MicroRNAs (miRNAs) are small (21–24 nucleotides), non-coding RNAs that modulate gene expression in eukaryotes. Inside the nucleus, miRNAs are transcribed by RNA polymerase II as precursor RNAs, known as primary miRNAs (pri-miRNAs)^10^. Subsequently, they are processed by DICER-LIKE 1 (DCL1) protein to release the pre-miRNAs. DCL1 also carries out the subsequent cleavage of pre-miRNAs, upon which the miRNA/ miRNA* duplex is released and subsequently methylated at the 3′ terminus by Hua Enhancer 1 (HEN1). Further on, it is exported to the cytoplasm, by the plant exportin protein HASTY^10,11^. In the cytoplasm, miRNA/miRNA* duplex is separated and the guide strand is loaded into the RNA-induced silencing complex (RISC) through binding with Argonaute (AGO) proteins. This fully assembled RISC, which includes additional proteins such as Heat Shock Protein 90 (Hsp90), binds to the target transcript through sequence complementarity with its mature miRNA strand, to further direct either mRNA cleavage or translational inhibition^12,13^.

MiRNAs are well-known for their role in regulating various plants processes under biotic and abiotic stresses. Many studies have confirmed that abiotic stress conditions can induce an enormous production of miRNAs in plants^14,15^. In recent years, high-throughput sequencing and computational approaches have been used for identifying a large number of stress-related miRNAs. For example, in a study of Zhang *et al*.^16^, high-throughput sequencing analyses revealed cold stress-induced upregulation of 31 miRNAs in tea (*Camellia sinesis* (L.) Kuntze) plants. Similarly, Cao *et al*.^17^ studied the chilling stress response in wild tomato (*Solanum habrochaites*) and found that 192 miRNAs showed increased expression. Recently, Yang *et al*. found a different regulation of 84 miRNAs under low temperature stress in *Solanum aculeatissimum*^18^, while Zhou M. and Tang W. have reported that overexpression of rice miRNA156 (osa-miR156) results in increased cell viability and growth rate under cold stress in *Arabidopsis*, pine, and rice^19^. In our previous study, conserved and novel miRNAs in *M. oleifera* seeds were identified using Illumina platform technologies^20^. However, results produced by our bioinformatics pipeline could have been inaccurate because of the unavailability of genome data at the time. Furthermore, there is no report on *M. oleifera* miRNAs response to cold stress. This study aims to identify the differential expression of conserved and novel miRNAs and their target genes in *M. oleifera* leaf, callus and cold stress treated callus by high-throughput small RNA sequencing. In addition, the miRNA repertoire of *M. oleifera* seed was re-evaluated in view of the recently published *M. oleifera* genome assembly^21^.

## Methods

### Callus production and cold stress treatment

*M. oleifera* seeds were deprived of their external envelope and sterilized by 1 min immersion in 70% ethanol. They were subsequently washed with sterile distilled water and soaked for 10 min in a 2.5% sodium hypochlorite solution with the addiction of one drop of Tween 20. After 3–4 washes with sterile distilled water, seeds were ready to be sown in magenta boxes filled with medium containing full strength Murashige and Skoog (MS) basal medium (pH 5.8)^22^, 3% sucrose and 0.6% agar, previously sterilized by autoclaving at 121 °C for 20 min. Seed cultures were maintained in a growth chamber in a 16/8 h light/dark cycle at 22 °C.

Leaves from one-month old *Moringa* plants were sliced in uniformed pieces of 0.5 cm^2^ in size. These pieces were placed with the abaxial page in contact with the substrate in magenta boxes filled with medium described above, supplemented with 0.5 mg/L of 2,4-dichlorophenoxyacetic acid. The culture was maintained in dark, in a growth chamber at 22 °C. Cold stress was applied to one-month old callus culture through the cultivation at 4 °C for 7 days. After the stress treatments, callus was reduced to powder using liquid nitrogen, pestle and mortar, and stored at −80 °C for subsequent analysis.

### Small RNA sequencing

Next generation sequencing experiments, comprising samples quality control, were performed by Genomix4life S.R.L. (Baronissi, Salerno, Italy). Indexed libraries were prepared from 1 μg of purified RNA with TruSeq SmallRNA Sample Prep Kit (Illumina, CA, USA) according to the manufacturer’s instructions. Libraries were quantified using the Agilent 4200 TapeStation (Agilent Technologies, CA, USA) and pooled to ensure that each index-tagged sample was present in equimolar amounts, with final concentration of 2 nM each. The pooled samples were subject to cluster generation and sequencing using an Illumina NextSeq. 500 System (Illumina, CA, USA) in a 1 × 75 single read format at a final concentration of 3 pmol.

### Pre-processing of sequencing data

Generated raw sequence files underwent quality control analyses using FastQC^23^. Adapter sequences were trimmed using Cutadapt^24^ with default parameters. The preprocessing tools included in the miRDeep2^25^ suite of analysis were used for converting files in FASTA format, discarding reads shorter than 18 nt and collapsing the duplicates.

### Prediction of high-confidence and novel miRNAs

mirDeep2^25^ was used to predict mature, star and precursor sequences for well-annotated and novel miRNAs. Additionally, the secondary structure, as well as a detailed aligned report, is produced for each predicted miRNA. In the analysis, the *M. oleifera* genome assembled by Tian *et al*.^21^ and *Arabidopsis thaliana* (L.) Heynh. miRNAs precursors list were used as references.

### Prediction of low-confidence miRNAs

Blast alignment^26^ (blastn version 2.6.0) respect to all plant-related, mature miRNAs annotated in miRBase^27^ (release 21), was performed with the following parameters: -*task blastn-short*, -*perc_identity* 80, -*word_size* 15 and -*evalue 1e*^*−*3^. Identified matches were named according to the most abundant miRNA isoform highlighted in the comparison.

### Evaluation of the conservation rate for low-confidence miRNAs, across all plant species

In a wide evolutionary context, known and low-confidence miRNAs from *M. oleifera* were compared with the whole plant-specific miRBase^27^ [including 4 Coniferophyta (*Cunninghamia lanceolata* (Lamb.) Hook., *Pinus taeda* L., *Pinus densata* Mast., *Picea abies* (L.) H.Karst.), 1 Bryophyta (*Physcomitrella patens* (Hedw.) Bruch & Schimp.) 1 Lycopodiophyta (*Selaginella moellendorffii* Hieron.) 1 ancestral Magnoliophyta (*Amborella trichopoda* Baill.), 50 Magnoliophyta dicotyledons (including species of Asteraceae, Brassicaceae, Caricaceae, Cucurbitaceae, Euphorbiaceae, Fabaceae, Lamiales, Linaceae, Malvaceae, Ranuncolaceae, Rhizophoraceae, Rosaceae, Rutaceae, Salicaceae, Solanaceae, Vitaceae family) and 12 Magnoliophyta monocotyledons (*Aegilops tauschii* Coss., *Brachypodium distachyon* (L.) Beauv., *Elaeis guineensis* Jacq., *Festuca arundinacea* Schreb., *Hordeum vulgare* L., *Oryza sativa* L., *Saccharum officinarum* L., *Saccharum* sp. L., *Sorghum bicolor* L. Moench, *Triticum aestivum* L., *Triticum turgidum* subsp. durum (Desf.) Husn., *Zea mays* L.) in order to compute the conservation frequency.

The conservation rate for a selected miRNA (c_i_) is mathematically defined as:

$${c}_{i}=\,\mathop{\sum }\limits_{n=0}^{k}[sequenc{e}_{i}=sequenc{e}_{n}]$$

where k = number of known plant-related miRNAs stored in miRBase^27^

### Prediction of miRNA targets

In order to predict the putative genes regulated by miRNAs identified in our samples, we took advantage of psRNATarget^28^. The cDNA library used is the TAIR v10 of *A. thaliana*, while the scoring scheme follows the V2, released in 2017 by the authors.

### Gene Ontology (GO) enrichment analysis

To better understand the biological functions underlying target genes, we performed enrichment analyses on the three categories of GO (biological processes, molecular functions, cellular compartments). GO annotation of the predicted targets was performed by uploading the list of highlighted genes in the PANTHER classification system^29^.

### Experimental validation of miRNAs using quantitative real-time PCR analysis

Total RNA was extracted from *M. oleifera* leaves, callus and cold stress treated callus using the NucleoSpin miRNA kit (Macherey-Nagel GmbH&Co., Germany), according to the manufacturer’s protocol. Quality and quantity of the total RNA were evaluated by Agilent 2100 Bioanalyzer (Agilent Technologies, CA, USA) and by spectrophotometry (NanoDrop 2000, ThermoFischer Scientific, USA), respectively.

cDNA synthesis was performed with a miScript II RT Kit (Qiagen, USA) from 50 ng of sample. A quantitative real-time PCR analysis of mol-miR159a, mol-miR167a, mol-miR168a, mol-miR156a, mol-miR395a, mol-miR162a, mol-miR166i, mol-miR160h, mol-miR398b, mol-miR396a was performed using miScript SYBR Green PCR Kit according to the manufacturer’s protocol (Qiagen, USA) and the reaction was performed using a Rotor-Gene Q (Qiagen, USA) machine. The amplification conditions were: activation/denaturation at 95 °C for 10 min followed by 40 cycles of denaturation at 94 °C for 10 sec, annealing at 60 °C for 30 sec and extension at 70 °C for 30 sec. All reactions were performed in triplicate for each sample and 5S rRNA was used as the internal control gene. Relative expression levels of miRNAs were quantified by using the 2^−ΔΔCt^ method as widely described in our previous work^30^.

## Results

### Construction and sequencing of small RNA libraries

**Figure 1:**
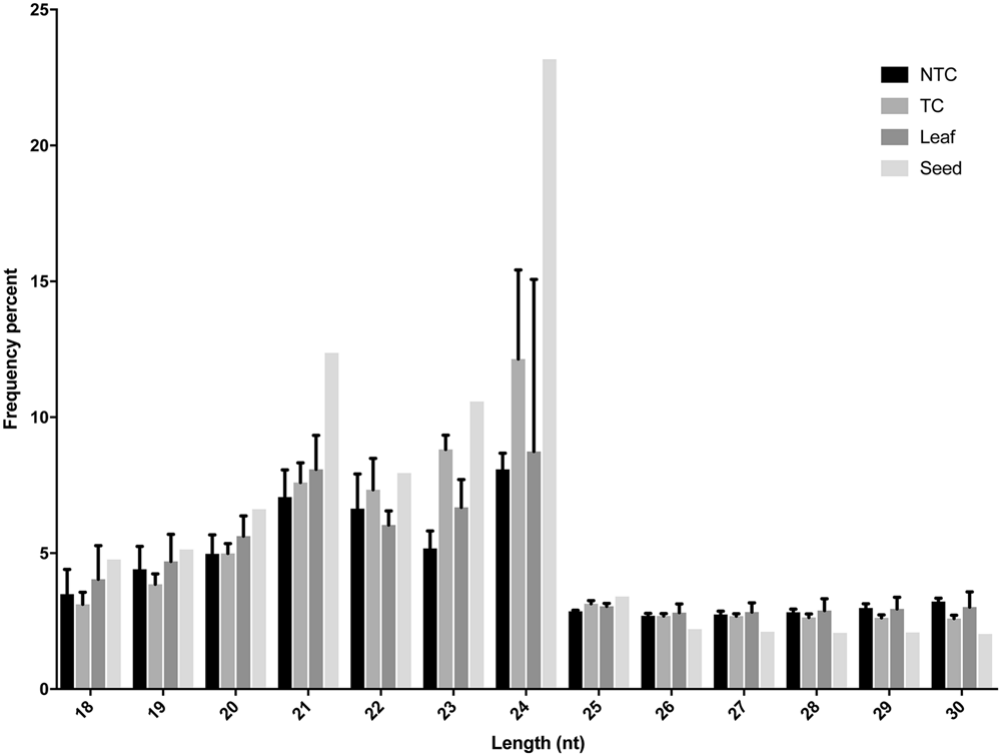
The length distribution of the clean and unique reads from leaf, seed and callus of *M.oleifera*. NTC: Non Treated Callus. TC: Treated Callus.

To identify *M. oleifera* miRNAs, total RNA was extracted from leaf, callus (Non Treated Callus; NTC) and cold stress treated callus (Treated Callus; TC) in order to construct nine small RNA libraries. Next, the libraries were sequenced using Illumina NextSeq. 500 System sequencing platform (Illumina, CA, USA) and analysed by bioinformatics approach. A total number of 71,900,401 raw reads was obtained from leaf (3 replicates): 72,962,350 from NTC (3 replicates) and 69,876,775 from TC (3 replicates). After removing the adaptors, low quality reads and contaminants, 63,698,781 clean reads from leaf, 71,445,523 from NTC and 68,889,789 from TC were obtained. As already reported in our previous work^20^ the number of raw reads for seed tissue before and after cleaning process, was 31,290,964 and 22,737,895 - respectively. The clean and unique reads were subsequently subjected to size distribution analysis as shown in Fig. 1. The filtering analysis reported the length of clean and unique reads of small RNAs to be between 21 to 24 nucleotides (nt). The 24 nt was the most abundant size in seed and leaf (23% and 8.7%, respectively) followed by 21, 23 and 22 nt in size. In both NTC and TC, the most frequent small RNAs were 24 nt in length (8% and 12%, respectively), followed by small RNAs of 23, 21 and 22 nt in length, in TC and of 21, 22 and 23 nt in length in NTC.

### Known miRNAs in M. oleifera

**Figure 2:**
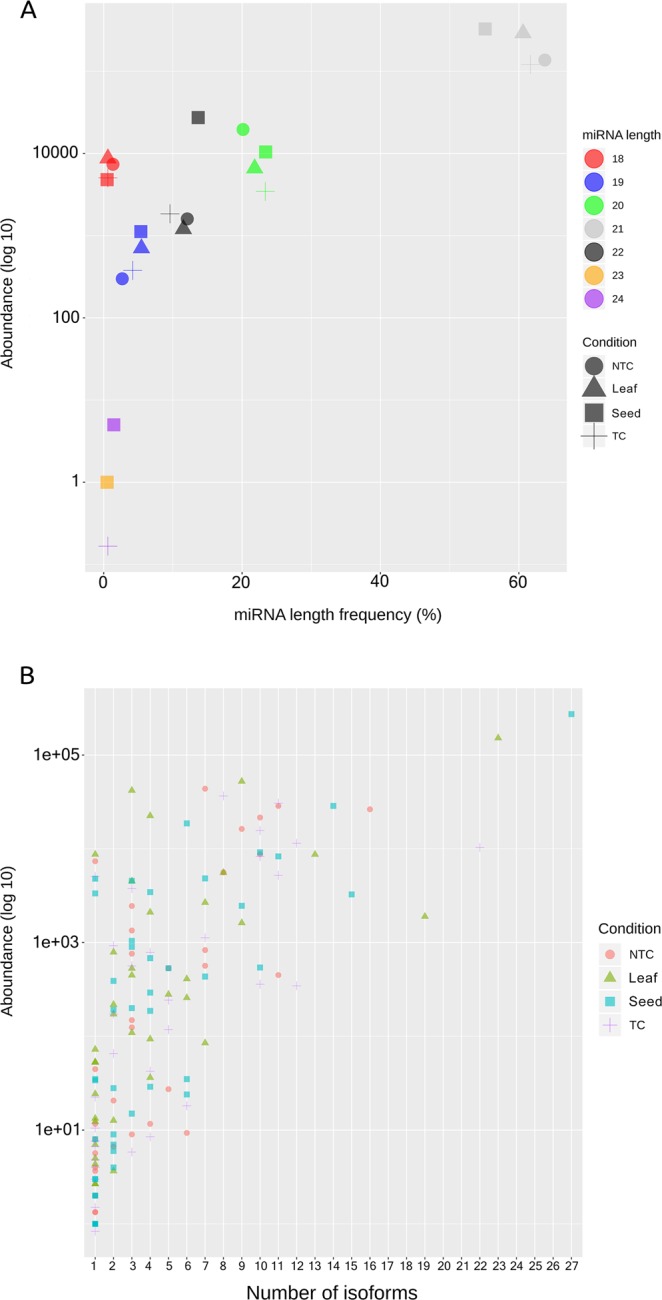
Size distribution vs abundance (**A**) and Number of isoforms vs abundance (**B**) in all the smallRNA libraries. The analysis was performed considering high and low confidence conserved smallRNAs in accordance for all experimental conditions. NTC: Non Treated Callus. TC: Treated Callus.

As for the length distribution of mature miRNAs is concerned (high and low confidence miRNAs), 21 nt is the most frequent class with the majority reads in all samples, followed by 20, 22, 19 and 18 nt. The majority of miRNAs from leaf belongs to 21 nt category (61%, 2.9*10^5 total number of reads). The abundance of reads supporting this length is notably higher also in seed (55.1%) with a great number of reads (3.2*10^5). NTC and TC presented a highly frequent 21 nt miRNAs size (63.8% and 61.7%, respectively) with a high number of reads (1.3*10^5 and 1.2*10^5, respectively) (Fig. 2A).

All the predictions obtained by the analysis with mirDeep2 software^25^ were classified as high-confidence. Table 1 shows the most abundant high-confidence miRNAs (over 100 reads) in all experimental conditions. For each of them, a detailed set of sequence (mature, star, precursor) and structural information is reported in Supplementary Table 1. As for the expression of high-confidence miRNA isoforms, the most abundant in NTC is mol-miR398c_1 with 4.3*10^4 reads (mean value of triplicate). In TC, mol-miR398c_3 is the most frequent isoform with 3.1*10^4 reads (mean value of triplicate) while in leaf and seed, mol-miR166u_5 and mol-miR166u_2 with 1.6*10^5 and 1.3*10^5 reads (mean value of triplicate), respectively, are the most abundantly expressed isoforms (Supplementary Table 1).

**Table 1:** Summary of the most abundantly expressed miRNAs in different experimental conditions. Data are reported as the sum of identical mature sequence isoforms. NTC: Non Treated Callus. TC: Treated Callus.

miRNA family	miRNA name	Sequence	Read Counts			
			NTC	TC	LEAF	SEED
**High confidence miRNAs**						
156	156f^*1,2,4*^	TGACAGAAGAGAGTGAGCAC	399	693	589	2,427
	156f^3^	TGACAGAAGAGAGTGAGCA	0	0	331	0
	156t^1*-3*^	TTGACAGAAGAAAGAGAGCAC	0	0	99	126
157	157a^1*-8*^	TTGACAGAAGATAGAGAGCAC	1,797	674	64,679	1,044
160	160h^*1-5*^	TGCCTGGCTCCCTGTATGCCA	43	26	446	0
162	162a^*1-4*^	TCGATAAACCTCTGCATCCAG	206	284	351	176
166	166e^*1-5*^	TCGGACCAGGCTTCATTCCCC	8,026	10,774	55,612	65,352
	166i^*1*,2^	TCGGACCAGGCTTCATTCCCC	8,021	10,752	55,668	65,306
	166u^1*-5*^	TCTCGGACCAGGCTTCATTCC	25,234	24,900	202,663	137,652
167	167d^1*-6*,1*1*^	TGAAGCTGCCAGCATGATCTGA	203	135	297	7,449
	167d^*7-10,12,13*^	TGAAGCTGCCAGCATGATCT	115	199	311	0
	167h^*1-3*^	TGAAGCTGCCAGCATGATCTT	141	124	1,060	18,461
171	171a^*1-9*^	TGATTGAGCCGTGCCAATATC	0	7	7,464	459
319	319 ^*1*^	TTGGACTGAAGGGAGCTCCCT	0	7,082	0	0
	319 ^*2,3*^	TTGGACTGAAGGGAGCTCCC	17,830	10,551	2,543	1,158
	319e^*1*^	TTGGACTGAAGGGAGCTCC	197	376	25	0
	319e^*2*^	TTTGGACTGAAGGGAGCTCCT	0	0	14	983
	319e^*3*,4^	TGGACTGAAGGGAGCTCC	215	0	0	0
	319g^1^	TGGACTGAAGGGAGCTCC	103	0	0	0
390	390a^1*-5*^	AAGCTCAGGAGGGATAGCGCC	4,251	11,710	370	3,471
	390d^*1-4*^	AAGCTCAGGAGGGATAGCGCC	1,336	6,668	114	1,171
393	393a^*1-*2^	TCCAAAGGGATCGCATTGATC	75	101	89	1,301
394	394b^1*-3*^	TTGGCATTCTGTCCACCTCC	21	60	2,102	3,444
395	395a^1^	GTTCCCCTGAGCACTTCACC	0	0	0	257
396	396h^*1*^	TTCCACAGCTTTCTTGAACTG	8,050	12,762	3,256	891
397	397a^*1*,2^	TCATTGAGTGCAGCGTTGATG	20,281	14,614	777	631
398	398c^1*-3*^	TGTGTTCTCAGGTCGCCCCTG	43,595	88,821	22,492	3,343
399	399a^1*,2,5-8*,11,1*2*^	CGCCAAAGGAGAGTTGCCCTT	90	195	130	0
	399a^*3,4*^	CGCCAAAGGAGAGTTGCCCT	0	0	0	290
	399a^*9*,1*0*^	CGCCAAAGGAGAGTTGCCC	0	3	130	0
	399d^*5,7*^	TGCCAAAGGAGAGTTGCCCTT	0	0	12	103
535	535a^1*-3*^	TGACAACGAGAGAGAGCACGC	65	310	853	389
	535a^*4*^	GTGCTCTCTATCGTTGTCA	0	0	122	0
827	827 ^1^	TTAGATGACCATCAACAAACG	735	1047	12	27
**Low confidence miRNAs**						
156	156^1^	TTGACAGAAGATAGAGAGC	12	12	121	8
	156a^*2*^	TGACAGAAGAGAGTGAGCAC	0	0	104	0
	156c^1^	TTGACAGAAGAAAGAGAGCAC	22	18	0	473
	156e^1^	TTGACAGAAGAGAGTGAGCAC	117	139	350	87
	156g-3p^*2*^	GCTCTCTATTCTTCTGTCATC	0	0	122	0
	156z^1^	TGACAGAAGATAGAGAGCAC	7	4	755	14
159	159^1^	TTTGGATTGAAGGGAGCTCTA	27,752	31,510	51,769	9,022
	159b-3p^1^	TTTGGATTGAAGGGAGCTCTT	108	143	471	63
	159c-3p^*1*^	CTTGGACTGAAGGGAGCTCCC	737	901	52	22
164	164a^*1*^	TGGAGAAGCAGGGCACGTGCA	7	9	7	180
166	166a^*1*^	TCGGACCAGGCTTCATTCC	84	54	106	666
	166a-5p^*1*^	GGAATGTTGTCTGGCTCGAGG	41	62	23	204
	166b^*1*^	TCGGACCAGGCTTCATTCCC	186	182	143	319
	166d^*1*^	TCGGACCAGGCTTCATTCCCG	58	105	445	554
	166e^*1*^	GGACCAGGCTTCATTCCCC	1	1	4	376
	166e-3p^*1*^	CTCGGACCAGGCTTCATTCCC	32	87	18	1,271
	166m^*1*^	CGGACCAGGCTTCATTCCCC	1	8	6	1,527
	166u^*1*^	TCTCGGACCAGGCTTCATTC	24	19	149	172
167	167-3p^*1*^	AGATCATGTGGCAGTTTCACC	0	0	0	247
	167b^2^	TGAAGCTGCCAGCATGATCTGG	0	2	277	33
	167b^3^	TGAAGCTGCCAGCATGATCTGA	0	0	3	132
	167d^1^	TGAAGCTGCCAGCATGATCTTA	30	33	200	2,237
168	168^1^	TCGCTTGGTGCAGGTCGGGA	34	83	21	139
	168a^1^	TCGCTTGGTGCAGGTCGGGAA	108	235	66	332
171	171-5p^1^	TATTGGCCTGGTTCACTCAGA	1	1	463	64
319	319a^1^	TTGGACTGAAGGGAGCTCCCT	3,480	2,146	101	150
	319a^3^	TTGGACTGAAGGGAGCTCCC	0	2,616	0	0
	319p^1^	TTTTGGACTGAAGGGAGCTCC	2	2	1	166
390	390a-3p^2^	CGCTATCCATCCTGAGTTTCA	0	113	1	27
	390b-5p^1^	AGCTCAGGAGGGATAGCGCC	7	136	9	169
393	393-5p^1^	TTCCAAAGGGATCGCATTGAT	6	8	7	172
	393b^1^	TCCAAAGGGATCGCATTGATCT	324	674	204	9,745
	393b-5p^1^	TTCCAAAGGGATCGCATTGATC	421	705	150	7,445
396	396-5p^1^	TTCCACAGCTTTCTTGAACT	518	892	223	267
	396a-3p^1^	GTTCAATAAAGCTGTGGGAAG	37	124	28	2
	396b^1^	TTCCACAGCTTTCTTGAACTT	106	238	5,033	7,061
	396e-3p^1^	CTCAAGAAAGCTGTGGGAGA	2	4	113	41
397	397b^1^	TCATTGAGTGCAGCGTTGATGT	494	449	14	48
398	398f^1^	GGTGTTCTCAGGTCGCCCCTG	61	103	25	0
403	403^1^	TTAGATTCACGCACAAACTCGT	70	117	123	85
	403a^1^	TTAGATTCACGCACAAACTCG	2,385	4,965	4,403	4,469
408	408^4^	ATGCACTGCCTCTTCCCTGGC	0	2,278	0	171
	408d^1^	TGCACTGCCTCTTCCCTGGC	465	944	198	27
530	530^1^	TCTGCATTTGCACCTGCACCT	4	9	441	893
6300	6300^1^	GTCGTTGTAGTATAGTGG	7,374	2,741	8,719	4,809

A subsequent secondary comparison between the sRNA sequences of ten libraries and known mature miRNA sequences from other plants was performed. The outcome of this analysis generated a total of 300 low-confidence miRNAs that share their mature sequence with miRBase (Release 21)^27^ records. All of them belong to a total of 56 miRNA families (Table 1, Supplementary Table 2). In most families, more than one precursor was identified. The most abundant isoforms resulted to be mol-miR159_1 in NTC, TC and leaf with 2.7*10^4; 3.1*10^4 and 5.1*10^4 reads (mean value of triplicate), respectively, and mol-miR393b_1 in seed with 9.7*10^3 reads (mean value of triplicate) (Supplementary Table 2).

In high and low confidence miRNA families, mol-miR166 showed a higher number of isoforms in seed (27 isoforms, 2.7*10^5 total number of reads), in leaf (23 isoforms, 1.5*10^5 total number of reads) and in TC (22 isoforms, 1.0*10^4 total number of reads), while in NTC, miRNA166 families possessed 16 isoforms with high abundance (2.6*10^4 total number of reads). Thirty-six percent of miRNA families has only one condition-specific isoform: among these, mol-miR6300 has the higher number of reads in leaf (8,719 reads), callus (7,374 and 5,057 reads in NTC and TC, respectively) and seed (4,809 reads) while mol-miR398 showed high expression only in seed (3,343 reads) (Fig. 2B).

**Figure 3:**
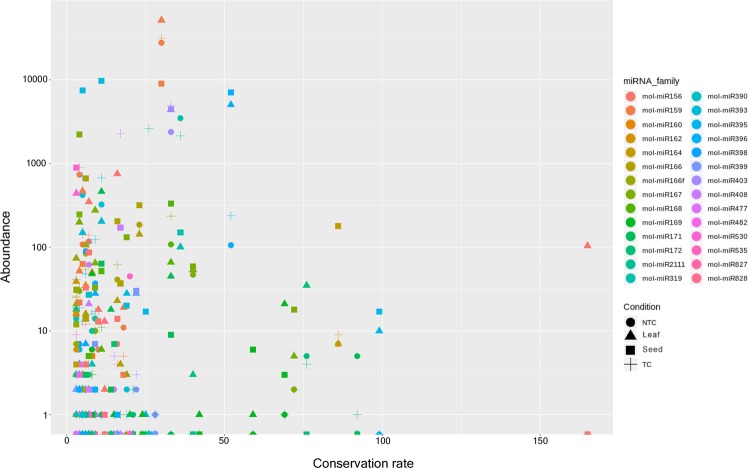
Conservation rate vs abundance of conserved miRNA families. The analysis was performed considering low confidence conserved top 100 miRNAs in accordance for all experimental conditions. NTC: Non Treated Callus. TC: Treated Callus.

As for low-confidence miRNAs, we assessed the correlation between conservation rate and abundance in all experimental conditions. mol-miR159_1 was the most abundantly expressed in *M. oleifera* leaf, NTC and TC (5.1*10^4, 2.7*10^4 and 3.1*10^4 reads respectively) with a low conservation rate among plants (rate 30). Mol-miR156a_2 was found to be the most conserved miRNA among plants (rate 165), although uncommon in all experimental conditions except for the leaf (104 reads). In addition, mol-miR395a_2, mol-miR171a_1, mol-miR164a_1, mol-miR172a_1, mol-miR167a_2 and mol-mir169b/l_1 showed a high conservation rate among plants and a relatively low abundance, except for mol-miR164a_1 in the seed. The remaining miRNAs, with the exception for mol-miR396b_1, showed a conservation rate below 50 with different abundance degree. (Fig. 3).

### Novel miRNAs in M. oleifera

A total of 392 novel microRNAs were predicted by identifying all the potential precursors (pre-miRNAs) and modelling their secondary structures. All the discovered sequences ranged in length between 18 and 25 nt (Supplementary Table 3). The sequences of most of the novel miRNAs were 21 nt long (102 sequences) while the second most abundant class was 18 nt long (69 seq). The lengths of novel miRNA precursors ranged from 37 to 105 nt, where the 43 nt was most abundant (17 seq). Leaf has the highest number of novel miRNAs (213 seq). NTC, TC and seed follow with 121, 85 and 56 sequences, respectively. The most abundant miRNA isoform was found in the leaf with 21 nt in length (mol-miR-n31/n211/n239; 7.8*10^5 reads), followed by seed (mol-miR-n323; 2.0*10^5 reads), TC (mol-miR-n217; 1.8*10^4 reads) and NTC (mol-miR-n290; 1.4*10^5 reads) with 20 nt in length.

### Identification of “cold-sensitive” miRNAs

**Figure 4:**
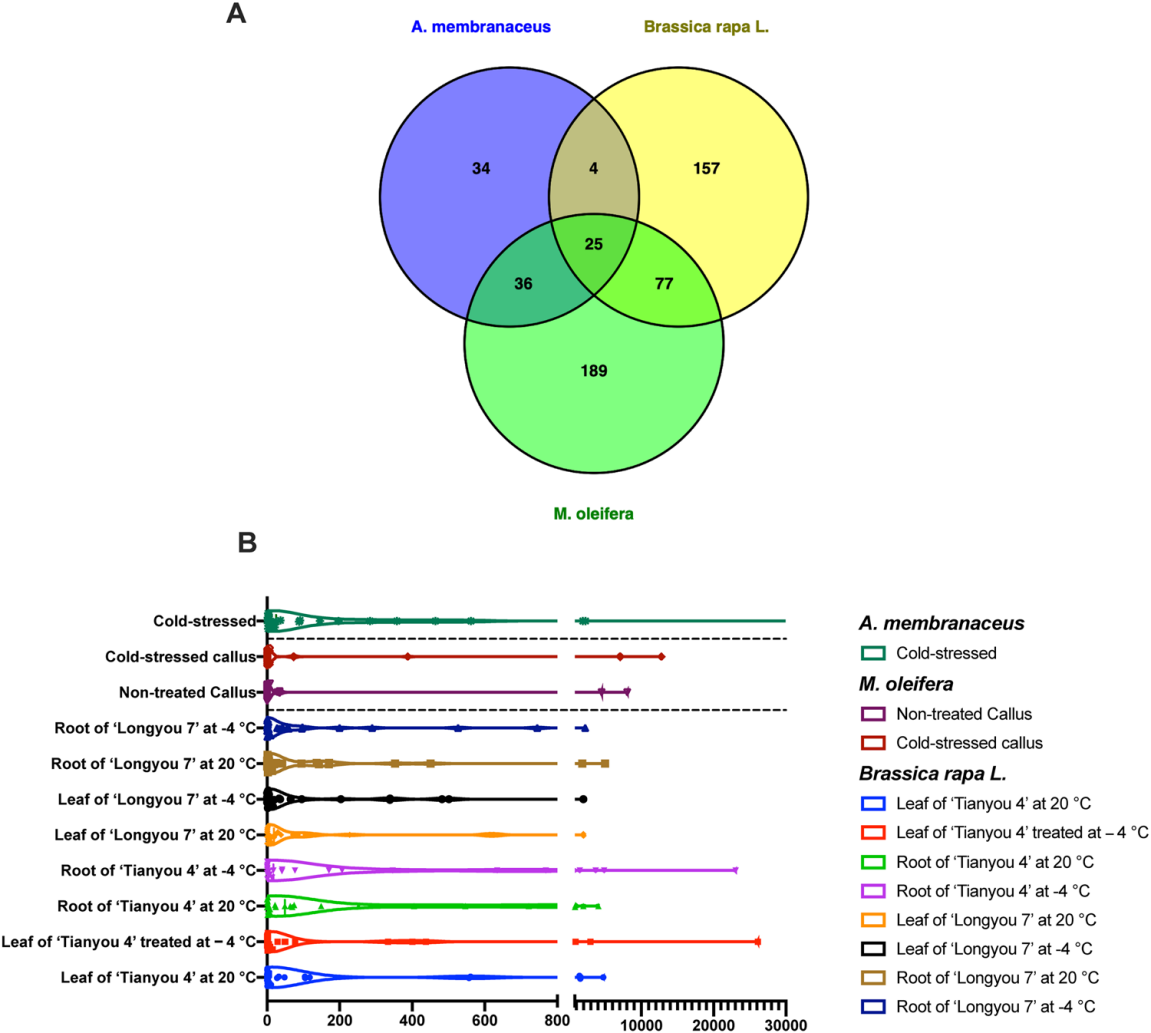
The number of miRNAs conserved across several cold-stress plants, is showed in a Venn diagram. (**A**) The mean abundance of this putative, “cold-responsive” miRNA signature across the different experimental conditions, is represented as a violin plot (**B**).

Cold stress has been shown to have an important influence on plant growth and development by modulation of gene expression. Some studies document that chilling stress increases the amount/slows down decomposition of polyphenols in some plants (*Nicotiana tabacum L*., *Vitis vinifera L*.)^31,32^. Moreover, chilling stress response induced increased expression of selected miRNAs in wild tomato (*Solanum habrochaites*)^17^, *Brassica rapa L*.^33^ and *Astragalus membranaceus*^34^. We performed a comparative analysis between the known miRNAs identified in *M. oleifera* and the ones published in the aforementioned studies. As shown in Fig. 4A, 25 miRNAs are conserved among the selected species. By looking at their global abundance within different conditions (Fig. 4B), it is not possible to determine whether the subset in its entirety is sensitive to the cold stress. However, miR-166e-3p in *Brassica rapa L*., mol-miR319_1 and mol-miR396h_1 in *M. oleifera*, ame-miR396-5 in *A. membranaceus* are upregulated after cold stress treatment.

### Identification of condition-specific and backbone miRNAs

**Figure 5:**
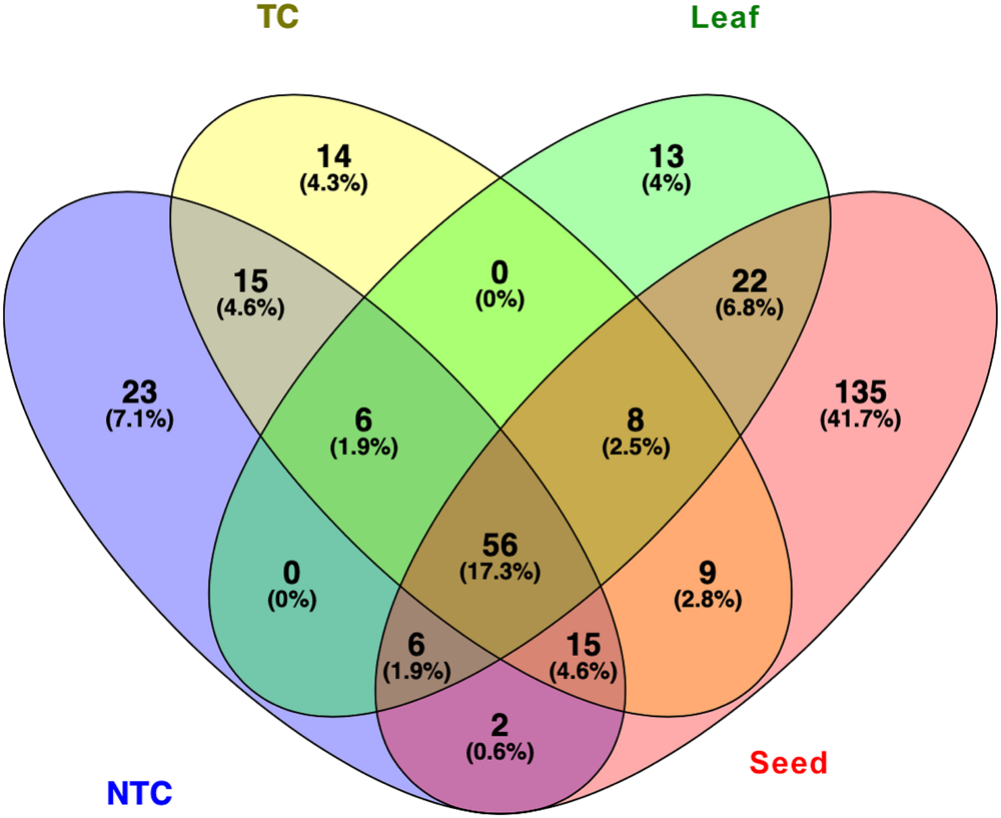
Venn diagram showing the number of conserved and novel miRNAs in accordance for all experimental conditions. NTC: Non Treated Callus. TC: Treated Callus.

In addition to analysis of the gene regulation mediated by miRNAs, the analysis of differential internal regulation of miRNA expression in different tissues and experimental conditions was performed. In plants, this fine-tuning mechanism is based on the regulation of miRNA’s decoy time^35^. As consequence, miRNA patterns in plants are modulated according to the tissue and the environmental conditions. On the other hand, we also defined a set of miRNAs whose expression is stable across different experimental conditions (backbone). In order to evaluate the modulation of miRNA patterns among tissues and cold-stress, we overlaid the high-confidence, low-confidence and novel miRNAs identified in each condition, separately (Fig. 5). A detailed list of tissue-specific and backbone miRNAs is also reported in the Supplementary Table 4. Among 56 miRNA families belonging to all experimental conditions, mol-miR166 and mol-miR159 families are the largest families identified with ten and eight members, respectively. A total of five different isoforms belongs to the mol-miR156 and mol-miR396 families. Several miRNAs appear to be tissue-specific: 135 unique miRNAs were found in the seed; among these, mol-miR166 (8 members), mol-miR167 (8 members), mol-miR156 (6 members) and mol-miR399 (6 members) were identified as well as 46 novel miRNAs.

### Prediction of miRNA targets

Plant miRNAs play important roles in diverse biological processes by cleaving target mRNAs or supressing their translation. In order to understand the biological functions of *M. oleifera* miRNAs, PNRD (plant non-coding RNA database)^36^ was used as a reference for the prediction of putative target genes for tissue-specific and backbone miRNAs. The analysis revealed 7,677 unambiguous target genes for 54 miRNA families conserved across all the experimental conditions (Supplementary Table 5).

The majority of target genes were transcription factors; these include key regulators of abiotic and biotic stress response, plant reproduction and plant growth/development gene such as *squamosa promoter-binding proteins* (*SPL* or *SBP*)^37,38^, *myb domain proteins* (*MYBs*)^39–41^, *ARM repeat superfamily protein*^42,43^*, Homeobox-Leucine Zipper Proteins* (*ATHBs*)^44,45^, *Auxin response factors* (*ARFs*)^46,47^, *Disease resistance protein*^48,49^*, F-box family protein*^50^ and *NAC domain transcription factors* (*NACs*)^51^.

Some of the identified miRNA targets are genes directly involved in resistance to biotic and abiotic stresses, including protein degradation (*ubiquitin carboxyl-terminal hydrolase 1*) and oxidoreductase activity (*ALDH22a1*).

Other predicted miRNA target genes are associated to plant growth/development, metabolism and plant reproduction; several genes are related to protein degradation (*auxin signaling f-box 2*), enzymatic activity (*laccase*, *RHOMBOID-like protein 1*, *clathrin heavy chain*. *zinc finger family protein, Hydrolase, Cysteine Protease, Cysteine/Histidine-rich C1 domain, Protein Kinase, NAD(P)-binding Rossmann-fold, Pectine lyase Kinase* activity, *RNA binding family protein, Methyltransferases* and *DEAD/DEAH box RNA helicase family protein*), plant development (*Pentatricopeptide repeat (PPR) superfamily protein, RING/U-box superfamily protein*), translation process (*Ribosomal protein*) and oxidoreductase activity (*2-oxoglutarate and Fe(II)-dependent oxygenase superfamily protein, Galactose oxidase)*.

### Gene Ontology (GO) enrichment analysis

The enrichment analysis of miRNA target genes represents a fundamental procedure for evaluating the miRNA-mediated biological modulations that occur in *Moringa* leaves, seed, TC and NTC. The terms belonging to GO biological processes (BP), molecular functions (MF), and cellular compartments (CC) have been enriched and the results for the top 20 terms plotted in a bar plot (Supplementary Figures 1_NTC, TC, LEAF, SEED). Analyses performed on targets for backbone miRNAs, identified certain biological functions and molecular processes that are crucial for the proper survival of the plant. On the other hand, the enrichment analysis of targets for condition-specific miRNAs highlights biological modulations that are essential in a specific, biological context.

#### Biological processes

In the leaf, the majority of miRNAs seems to be responsible for fine regulation of genes involved in leaf symmetry^52,53^; other genes regulate the development of different plant organs, such as anther^54^, floral whorl^55^, stamen^56^ and so on. In callus, most miRNAs are involved in positive heterochronic gene regulation of development, especially in stress conditions; other genes are involved in the biosynthesis and metabolic processes of beta-D-glucan^57^ (callus) and the lignin/phenylpropanoid catabolic processes^58^. In the seed, most frequently expressed genes regulate the maintenance of DNA methylation, negative regulation of intracellular signal transduction and specification of axis polarity^59^.

#### Cellular compartments

In leaf and seed, most of the regulations take place in the late endosome, vacuole and cell projection. In callus, most of the genes are localized in the 1.3-beta-D-glucan synthase complex (NTC) and mainly in the chloroplast endopeptidase Clp complex (TC). The presence of several genes located in the chloroplast could be correlated with the enrichment of stress-response genes. In fact, most of the transcriptional activities in the nucleus are regulated in part by signals derived from plastids. This process is named “retrograde signaling” and it is well amplified during responses to chemical, physical and biological stress^60^.

#### Molecular functions

Despite most of the enriched terms do not provide a detailed description of the involved molecular function, they clearly highlight the fine-tuning role of microRNAs in plants. Indeed, most of the targets are involved in xenobiotic transmembrane transporting ATPase activity^61^ (leaf), oxidoreductase and ATP-dependent peptidase activity^62^ (TC), superoxide dismutase copper chaperone activity^63^ (NTC), phosphorelay sensor kinase activity and xenobiotic transmembrane transporting ATPase activity^64^ (seed).

### Validation of conserved miRNAs by qRT-PCR

To confirm the expression pattern of the *M. oleifera* miRNAs, 11 conserved miRNAs with different expression profiles were randomly selected for real-time PCR analysis. The data collected demonstrate that the expression patterns are similar between the two analytical tools (Illumina sequencing and qRT-PCR) for six of the eleven miRNAs, whereas other show different expression pattern as detected between the two molecular tools.

**Figure 6:**
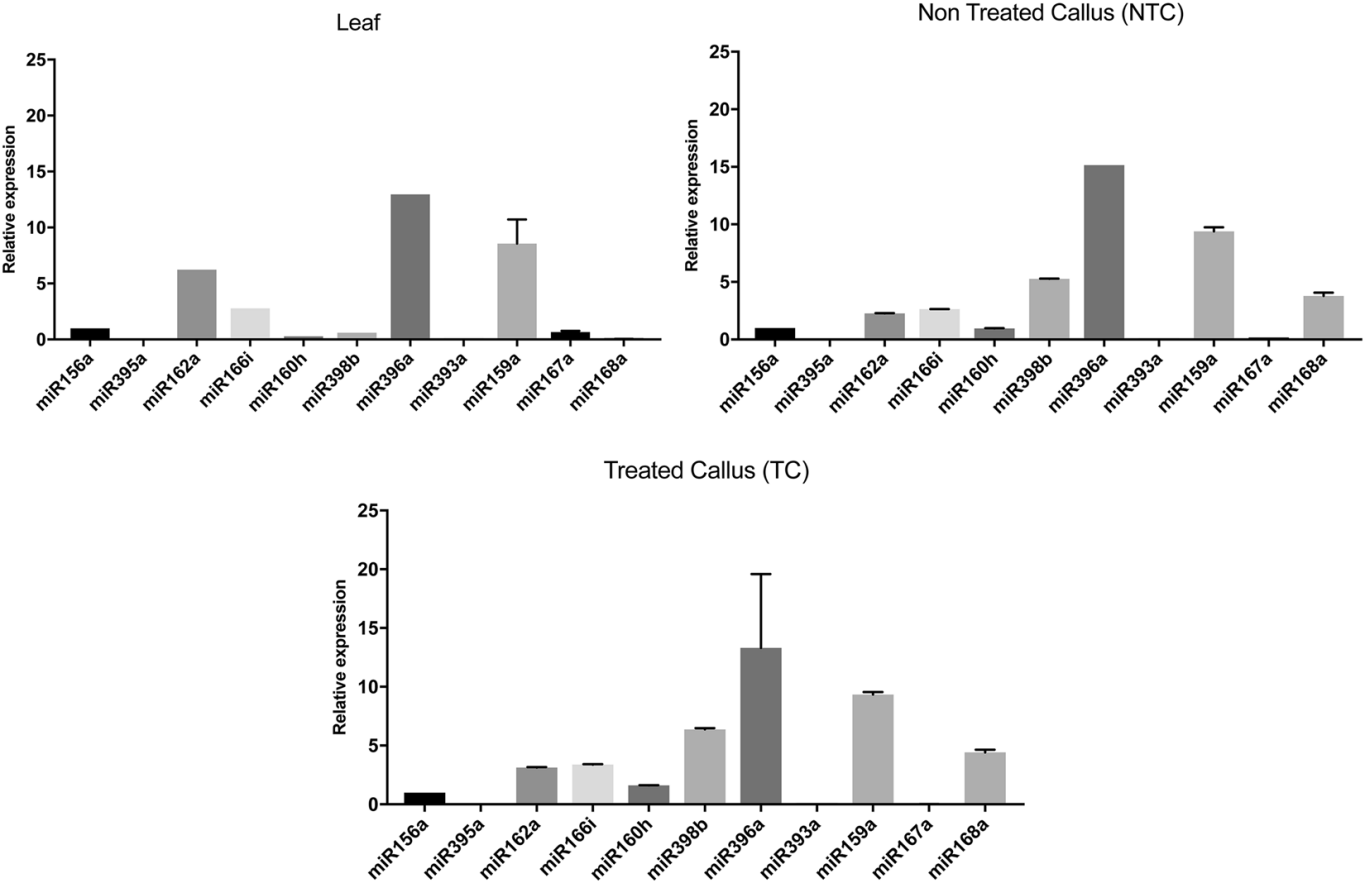
Quantitative RT-PCR analysis of conserved miRNAs in *M. oleifera* leaf and callus. The X axis represents different miRNAs. The Y axis represents the relative expression level of miRNAs. The 5S rRNA was used as an internal control. The expression level of miRNA156a was set as control and taken as 1 and expression level in all other miRNAs was quantified relative to it. The analysis was performed as triplicates, and the error bars indicate the standard deviations.

As illustrated in Fig. 6, mol-miR396a and mol-miR159a are more abundant than other miRNAs in leaf and callus (both NTC and TC). Mol-miR162a is more abundantly expressed in leaf than in callus (both NTC and TC), while mol-miR398b and mol-miR168a are more expressed in calluses. On the other hand, mol-miR166i and mol-miR160h show similar levels of expression in all tissues.

## Discussion

In the past decade, *M. oleifera* has gained growing attention for its nutraceutical and pharmacological functions as well as potential benefits for human health; several studies have demonstrated the great number of bioactive compounds contained in leaves, seeds, pods and flowers^1,2,4^. The large number of these bioactive compounds (such as secondary metabolites) might explain the pharmacological properties of this “miracle tree”. Recent studies have confirmed the high content of polyphenols (such as flavonoids and phenolic acids) in *M. oleifera* leaves^65^. These secondary metabolites are synthesized in the plant for its specialized need, in particular, ecological conditions; unlike primary metabolites, secondary metabolites are directly involved in the defence mechanism against environmental injuries. In human, *M. oleifera* polyphenols (such as flavonoids) are involved in protection against chronic diseases associated with oxidative stress, including cardiovascular disease and cancer^4^. Plant metabolic engineering has been used for enhancing biosynthesis of these pharmacological important phytochemicals^6–9^. One of the ways to increase polyphenol biosynthesis is by modulating levels of miRNAs – the ultimate regulators of biosynthesis and accumulation of secondary metabolites in plants^66–68^.

In our previous study^20^ we reported a high throughput sequencing of miRNAs from *M. oleifera* seed; some of them were involved in regulation of human genes when transfected into Hepatoma cell lines (HepG2)^69^. Being the part of the plant that contains the highest number of bioactive compounds, the leaf was chosen as model tissue for high-throughput sequencing in this study. Moreover, callus cells, given their undifferentiated nature, represent an optimal model for the identification and quantification of miRNA types present in *M. oleifera* in its basal condition. Low temperature was chosen as appropriate stress condition in the experimental procedure, considering that, due to natural geographical distribution of *M. oleifera*, this plant hardly ever comes into contact with this type of adverse condition. Indeed, cold stress has been shown to have an important influence on plant growth and development by modulation of gene expression. Despite a preliminary, explorative analysis did not highlight the presence of a “cold-sensitive” signature of microRNAs, specific isoforms were up-regulated in different plants. The application of the cold stress to a callus for further induction of somaclonal variations, could open, in turn, the possibility to develop a *M. oleifera* cell line better adapted to a temperate climate. For these reasons, *M. oleifera* calluses both untreated and exposed to abiotic stress seemed to be an interesting candidate models for this study.

The recent availability of data for the *M. oleifera* genome, allowed to re-evaluate our previous predictions from seeds and to identify a set of high-confidence and novel miRNAs from cold-stressed and non-stressed callus and leaf. A subsequent sequence alignment (Blast) compared to all plant-related mature miRNAs annotated in miRbase, allowed for identification of an additional set of low-confidence miRNAs. In the present study, a total of 131 known high-confidence miRNA isoforms (21 miRNA families), 300 known low-confidence miRNA isoforms (56 miRNA families) and 392 novel miRNA isoforms were identified in *M. oleifera* leaf, seed and callus with or without abiotic stress in the ten libraries. The majority of the identified miRNAs were 21 nt in length (60%; mean value of all experimental conditions), which is the canonical size for miRNAs generated from DCL1 processing. This result is similar to that of the conserved miRNAs predicted in *B. oleracea*^70^ and other plant species such as soybean^71^, maize^72^, switchgrass^73^ and Chinese cabbage^74,75^. In our study, the number of reads of the conserved 21 nt miRNAs is very high, especially for the leaf (4.6*10^5 reads). The result was consistent with our previous reports in which sequence length 21nt has the highest number of miRNA reads in *M. oleifera* seeds.

Mol-miR166 and mol-miRNA156 families contain larger number of isomiRs in the seed, leaves and in the treated callus; these microRNAs are involved in many biological processes including leaf development^76^, apical dominance^77^, floral transition and development^78^. MiRNA166, together with miRNA165, belong to another important class of miRNAs involved in *Shoot Apical Meristem (SAM)* maintenance. These two miRNAs share the same function of targeting/repressing *class III Homeodomain-Leucine Zipper (HD-ZIP III)* expression. These miRNAs play an important role in meristem maintenance, adaxial identity of leaves, lateral root growth and procambium identity.

The mol-miR156 family was a largely conserved miRNA family, followed by mol-miR395, mol-miR164 and mol-miR172. In our analysis, mol-miR156 family shows higher expression in leaf compared to other experimental conditions; these miRNAs belong to the largest families highly conserved in all land plants^79^. Previous studies demonstrated their role in SPL pathway regulating plant development, flowering and plastochron length.

In plants, many miRNAs seem to be universally expressed among diverse species, such as miRNA156, miRNA157, miRNA159, miRNA160, miRNA161, miRNA171, and so on.

However, there is a large number of miRNAs present in only few other species. For instance, mol-miR6300 was discovered in soybean and subsequently identified in Finger millet and in Chickpea, as well as *M. oleifera* seed (as reported in our previous work^20^). In flowers and leaves of Chickpea, this miRNA was found abundantly expressed; however, as predicted by other authors, its annotation in miRBase is ambiguous and needs experimental confirmation^80^. In our analysis, all the experimental conditions (especially for leaf) showed an up-regulation of this miRNA compared to other miRNAs. It would be interesting to investigate in more detail the biological function of this miRNA considering that it is highly expressed in many parts of this medicinal plant.

In this study, mol-miR159, mol-miR393 and mol-miR396 were found highly expressed in all experimental conditions. These results are in line with different studies, comprising various plant species, in which miRNA159 was shown to be very frequently detectable miRNA involved in fundamental plant biology roles, such as plant growth, and development^81^. In *A. thaliana*, miRNA159 has been shown to regulate anther and silique development by targeting MYB33. Mutation of the miRNA159-binding site on MYB33 mRNA resulted in pleiotropic defects including severely impaired fertility, stunted anthers, small siliques, and small seeds. Interestingly, miRNA159 accumulation was up-regulated by gibberellin (GA) application and GA-deficient mutants showed low miRNA159 accumulation. Treating these mutants with GA was itself sufficient to increase the accumulation of miRNA159 to wild-type levels and above, demonstrating the interplay between miRNAs and hormones in plant development^82^.

It is noteworthy to mention that miRNA159 is involved in cross-kingdom regulation of mammalian gene expression; Chin *et al*. found that plant miRNA159 could be detected in human sera and its levels were inversely correlated with breast cancer incidence and progression targeting human *Transcription Factor 7 (TCF7)* gene^83^.

Gene targets were computationally predicted for both conserved and novel miRNAs in order to elucidate the biological functions of miRNAs in *M. oleifera* leaf, seed and callus. As demonstrated by other studies, mol-miR159, very common in all experimental conditions, was predicted to target *MYB* transcription factors; these target genes have been reported to play an important role in abscisic acid (ABA) signalling during *A. thaliana* seed germination^84^. Moreover, mol-miR159 and its target gene (*GAMYB*) were involved in modulation of grapevine floral development in response to gibberellin (GA) treatments and this interaction has important implications for the molecular breeding of high-quality seedless grapevine berry. Mol-miR393, more common in seed than in leaf and callus, was shown to be involved in regulating various aspects of plant growth and development; for instance, mol-miR393 targets the *Transport Inhibitor Response 1* (TIR1) and *Auxin Signaling F-Box (AFB)* genes, involved in flower development thus contributing to the maize grain filling rate by regulating maize growth, development and environment stress response^85^. According to our analysis, this miRNA regulates enzymes involved mainly in Hydrolase and Methyltransferase activity including transcriptional regulation. *Growth-Regulating Factors (GRF)* genes are targeted and regulated by mol-miR396, very common in *M. oleifera* seed and leaf. In *A. thaliana* leaves in phase of development, expression of miRNA396 in the distal region of the leaf blade restricts GRF activity, thereby confining cell proliferation to the proximal leaf blade. As the leaf matures, miRNA396 level increases in the developed leaf leading to decreasing levels of GRF, stopping leaf blade growth. This conserved miRNA and its targets produced similar result in our analysis. Our results showed a very high conservation rate for mol-miR156 and a moderate abundance in *M. oleifera* leaf. This miRNA suppresses *SPL* and represses flowering in many plant species. As reported by various authors, the miRNA156 family is one of the largest miRNA gene families and members of the family are highly conserved in all land plants.

The predicted potential targets of *M. oleifera* novel miRNAs were involved in different molecular functions such as transcription factor activity, Auxin signalling and Ethylene responses. Many of these target genes are involved in plant growth and development, or in stress responses. Furthermore, our predicted analysis found a novel miRNA (mol-miR-n111) overexpressed in treated callus respect to non-treated callus that regulate many target genes, including *MYB* activity; previous reports have indicated that this transcription factors is also a target of miRNA159^86,87^ In our analysis, mol-miR159 was up-regulated in all experimental conditions; in plants, MYBs are involved in different processes including primary and secondary metabolism, cell fate and identity, development, and responses to biotic and abiotic stresses including cold tolerance. The novel mol-miR-n323, commonly expressed in seed, was predicted to regulate different target genes, including *Histone Deacetylase 5 (HDA5)*. In *A. thaliana*, this protein is present in a protein complex involved in the regulation of flowering time^88^; it’s regulated by mol-miR482, uncommon in seed. In eukaryotes, epigenetic mechanisms (such as acetylation) play a crucial role in the regulation of gene expression; the HDACs protein complex has been associated with various developmental processes such as flowering, floral organ identity, seed development and circadian clocks.

In order to identify conserved and novel miRNAs involved in the biosynthesis of secondary metabolites in plants, we performed an overlaid analysis to identified specific miRNAs. Mol-miR408, more common in treated callus respect to others experimental conditions, are involved in the biosynthesis of benzyliso-quinoline alkaloids (BIA). This alkaloid is synthesized mainly by an agronomically and economically important medicinal plant, the Opium poppy (*Papaver somniferum* L.), used for main morphine alkaloid production^67^. In *Argemone mexicana* L. (Papaveraceae), BIA is used to treat different disorders, given its antimicrobial, antiparasitic, antimalarial, pesticide, cytotoxic and neurological properties^89^. Another important miRNA implicated in the biosynthesis of the secondary metabolites in plants is mol-miR156. This miRNA, targeting *SPL9* gene, is up-regulated in *M. oleifera* leaf and seed and seems to be involved in the Anthocyanin and Sesquiterpenoid/triterpenoid biosynthesis. Anthocyanins are flavonoids with antioxidant properties and can therefore can be used potentially as dietary nutraceuticals for human health. Data from several epidemiological studies have reported an inverse correlation between anthocyanin intake and risk of cardiovascular disease (CVD) or CVD-related mortality^90^. Different authors have shown a beneficial effect of anthocyanin rich foods on gut microbiota; the result of *in vitro* and *in vivo* studies highlighted a significant proliferative effect on *Bifidobacterium* spp., known for their wide use in probiotics and for the treatment of irritable bowel syndrome, as well as inhibition of *Clostridium histolyticum*, pathogenic in humans^91^. Mol-miR396b_1, upregulated in *M. oleifera* seed and leaf, is well known for being able to regulate the biosynthesis of Flavonol glycoside by target Kaempferol 3-O-beta-D-galactosyltransferase^92^. Dietary flavonoids isolated from different medicinal plants have received an increased attention due to their considerable benefits in the prevention and management of modern diseases such as cancers, diabetes, and cardiovascular diseases^93^.

In conclusion, this is the first comprehensive identification of conserved and novel miRNAs in *M. oleifera* leaf, seed and callus (stressed and non-stressed). This dataset represents an important supplement to the existing *M. oleifera* miRNA database and shall be useful in understanding the possible role for enhancing biosynthesis of pharmacologically important phytochemicals by plant miRNAs during biotic and abiotic stresses. Further studies are necessary in order to elucidate this complex regulatory network potentially able to improve human health in socially neglected populations by a “miracle” tree with high nutritional and medicinal value.

## Supplementary information

Supplementary Dataset 1

Supplementary Dataset 2

Supplementary Dataset 3

Supplementary Dataset 4

Supplementary Dataset 5

Supplementary Figure 1_TC

Supplementary Figure 1_SEED

Supplementary Figure 1_NTC

Supplementary Figure 1_LEAF

## Data Availability

The sequences from the small RNA library have been deposited in the Gene Expression Omnibus (GEO) database, the accession number is GSE119247 (https://www.ncbi.nlm.nih.gov/geo/query/acc.cgi?acc=GSE119247).
