# Publisher Correction: Identification of microRNAs and relative target genes in *Moringa oleifera* leaf and callus

**DOI:** 10.1038/s41598-019-55237-0

**Published:** 2019-12-04

**Authors:** Stefano Pirrò, Ivana Matic, Arianna Guidi, Letizia Zanella, Angelo Gismondi, Rosella Cicconi, Roberta Bernardini, Vittorio Colizzi, Antonella Canini, Maurizio Mattei, Andrea Galgani

**Affiliations:** 1 Mir-Nat s.r.l., Rome, 00133 Italy; 2 Bioinformatics Unit, Centre for Molecular Oncology, Barts Cancer Institute, Queen Mary University London, London, EC1M 6BQ UK; 3 Department of Biology, University of Rome Tor Vergata, Rome, Italy; 4 CIMETA, University of Rome Tor Vergata, Rome, Italy

Correction to: *Scientific Reports* 10.1038/s41598-019-51100-4, published online 22 October 2019

The original version of this Article contained errors in the spelling of the authors Stefano Pirrò, Ivana Matic, Arianna Guidi, Letizia Zanella, Angelo Gismondi, Rosella Cicconi, Roberta Bernardini, Vittorio Colizzi, Antonella Canini, Maurizio Mattei & Andrea Galgani which were incorrectly given as Pirrò Stefano, Matic Ivana, Guidi Arianna, Zanella Letizia, Gismondi Angelo, Cicconi Rosella, Bernardini Roberta, Colizzi Vittorio, Canini Antonella, Mattei Maurizio & Galgani Andrea respectively.

These errors have now been corrected in the PDF and HTML versions of the Article.

