# Supplementary material for: Identification of microRNAs and relative target genes in Moringa oleifera leaf and callus
Source: Sci Rep. 2019 Oct 22;9:15145. doi: 10.1038/s41598-019-51100-4 (PMC6805943; doi:10.1038/s41598-019-51100-4)

**Supplementary Figure 1_Non-treated Callus**. Gene ontology categories of predicted target transcripts of differentially expressed miRNAs in *M. oleifera*. Categorization of miRNA-target genes was performed according to the Biological Processes, Molecular Functions and Cellular Compartments.


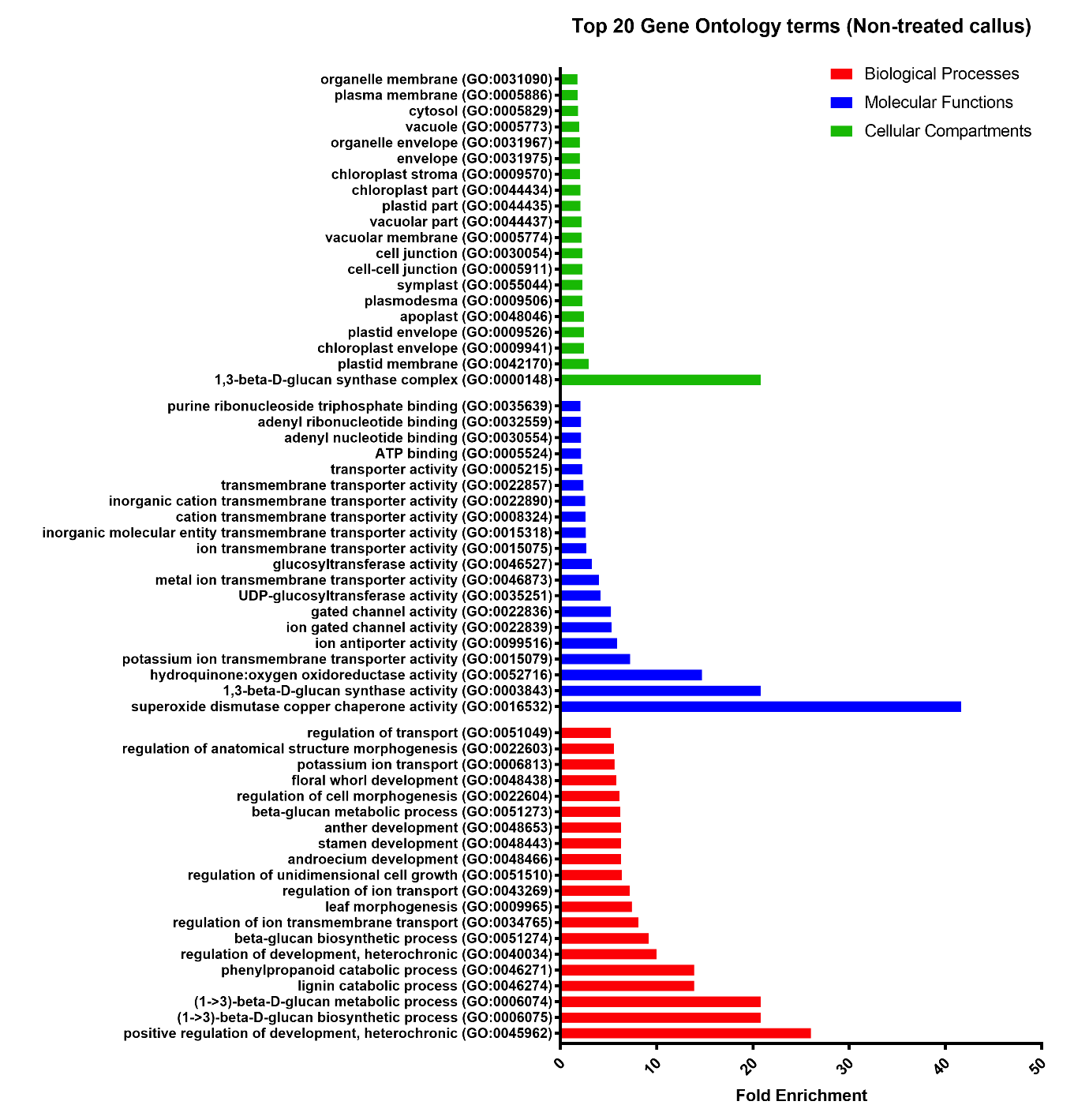

Supplement: Supplementary file 7 — Supplementary Figure 1_SEED [file 41598_2019_51100_MOESM7_ESM.docx]
